# Global gene expression profiling of healthy human brain and its application in studying neurological disorders

**DOI:** 10.1038/s41598-017-00952-9

**Published:** 2017-04-18

**Authors:** Simarjeet K. Negi, Chittibabu Guda

**Affiliations:** 1 Department of Genetics, Cell Biology and Anatomy, University of Nebraska Medical Center, Omaha, NE 68198 USA; 2 Bioinformatics and Systems Biology Core, University of Nebraska Medical Center, Omaha, NE 68198 USA; 3 Department of Biochemistry and Molecular Biology, University of Nebraska Medical Center, Omaha, NE 68198 USA; 4 Fred and Pamela Buffet Cancer Center, University of Nebraska Medical Center, Omaha, NE 68198 USA

## Abstract

Brain function is governed by precise regulation of gene expression across its anatomically distinct structures; however, the expression patterns of genes across hundreds of brain structures are not clearly understood. Here, we describe a gene expression model, which is representative of the healthy human brain transcriptome by using data from the Allen Brain Atlas. Our in-depth gene expression profiling revealed that 84% of genes are expressed in at least one of the 190 brain structures studied. Hierarchical clustering based on gene expression profiles delineated brain regions into structurally tiered spatial groups and we observed striking enrichment for region-specific processes. Further, weighted co-expression network analysis identified 19 robust modules of highly correlated genes enriched with functional associations for neurogenesis, dopamine signaling, immune regulation and behavior. Also, structural distribution maps of major neurotransmission systems in the brain were generated. Finally, we developed a supervised classification model, which achieved 84% and 81% accuracies for predicting autism- and Parkinson’s-implicated genes, respectively, using our expression model as a baseline. This study represents the first use of global gene expression profiling from healthy human brain to develop a disease gene prediction model and this generic methodology can be applied to study any neurological disorder.

## Introduction

Power of the brain arises from its hundreds of distinct structures and the orchestrated regulation of genes across them^1, 2^. It has been known that the expression profiles of genes in the brain are reasonably stereotyped between individuals^2, 3^. The recent availability of comprehensive expression data at high neuroanatomical resolution from sources like Allen Brain Atlas (ABA)^4^ has now made it possible to discover intricate expression patterns. Such data can be used to generate a profile of gene expression patterns that are consistent across healthy human brains in different individuals. We can then extend the application of these homogenous expression patterns as a baseline to predict new genes that may be implicated in neurological disorders by employing machine learning algorithms.

A number of studies have examined the global gene expression profiles in human central nervous system (CNS), but these comparisons were either between CNS and non-CNS tissues^5^ or between different species like humans and mice^6, 7^ or humans and non-human primates^8^. However, the anatomical structural differences and a large difference in size between the human and mouse brains limits the use of mice for understanding the human brain^6, 9, 10^. Also, the transcriptome profile of human brain differs significantly from that of other primates^11–14^. As for a handful of high-throughput transcriptome studies that use the human brain samples, they were conducted in pre-set anatomical areas of interest^1, 15^, which restrict the broader interpretation of global gene expression patterns. Additionally, meta-analysis of transcriptome studies is usually carried out by the amalgamation of datasets from multiple smaller studies conducted under different experimental conditions on grossly matched samples for neuroanatomical precision. The inconsistency resulting from such pooled samples can form a major shortcoming in cross-study analysis of data from multiple studies^9, 15, 16^.

In addition to understanding the healthy brain transcriptome, investigation of neurological disorders presents more unique challenges. Availability of diseased human brain tissue samples that are dissected at a high neuro-anatomical resolution continues to be a major issue. Therefore, very often multiple studies focus on using blood samples from the patients to investigate gene signatures in neurological disorders^17–21^. Even though the blood samples are easily accessible and can support large population- based collections, they do not accurately represent the expression profile of a patient’s brain^22^. To overcome this issue, researchers have attempted to induce pluripotent stem (iPS) cells from individuals with particular disorders and prompt the regeneration of specific neuronal cell types in order to study these *in-vitro* ^23^. However, the iPS technology is still in its infancy due to the challenges associated with low efficiency and high technical expertise requirement. Taken together, many studies have explored gene expression profiles in neurological disorders^20, 24, 25^, but none of them focuses exclusively on utilizing healthy tissue expression data from sources like ABA and exploring it in a framework of known disease implicated genes.

To directly address the dearth of knowledge in this area, we have used microarray data integrating the genomic and anatomic information from the ABA^2, 4^ and developed a model that recapitulates the gene expression patterns synonymously expressed in human brain across healthy individuals. We demonstrate here that gene expression data from multiple healthy individuals can be used to design an expression based model that accurately defines the consistent gene expression schematic of the brain transcriptome and can provide insights into the molecular functions and cross- talk between distinct brain regions. Using microarray data, we revealed statistically significant co-expression patterns with biological relevance, and by applying clustering we were able to show that distinct structures within a larger anatomical division can cluster together while still retaining their identity at the expression level. Our study also lays a foundation on how this knowledge can be used to complement the development of a prediction model for identifying potentially new candidate genes implicated in neurological diseases using machine learning. This methodology has a generic approach that can be applied to study other brain disorders like Autism, Schizophrenia, Alzheimer’s, etc.

## Results

### Generation of gene expression profile models

To identify genes with expression pattern sustenance across healthy adult human brains, we developed four different gene expression profile models using microarray data from the ABA^26^. The donors in ABA include five male and one female healthy individuals between the ages 24 to 57 years who died of natural causes. The profiles of these six donors showing their age, gender, ethnicity, handedness and the number of samples obtained per left hemisphere have been summarized in Supplementary Figure S1. For the purposes of data consistency, only the samples from left hemisphere of the brain were used for current study, as the right hemispheric microarray data were available for only two donors. About 400–500 tissue samples per left hemisphere were dissected for microarray data generation from each of the donors. Due to the normal variation in the brain size and morphology, some of the brain structures were sampled multiple times. Since these brain samples map back to the same neuroanatomical structural annotation, we averaged their expression values within each brain. Also, the brain structures that were sampled in at least two or more brains were considered to be part of the model. Finally, comparisons were made across a total of 216 distinct brain structures over 6 individuals. Note that throughout this manuscript, we will refer to the bigger conventional brain components such as cerebellum, hypothalamus, etc., as ‘brain regions’ and the smaller sub-anatomical components of these regions as ‘brain structures’ for consistency.

**Figure 1:**
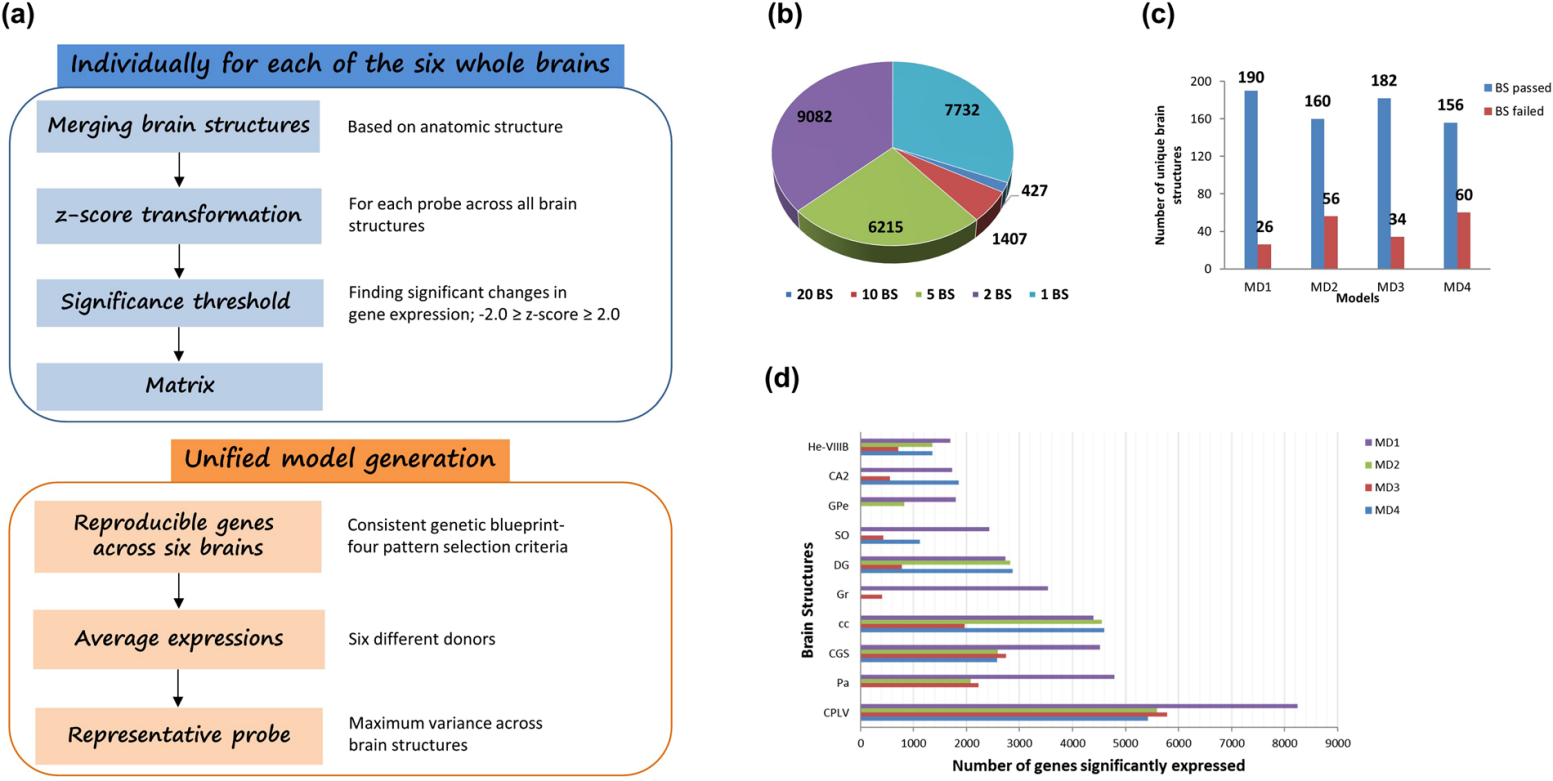
(**a**) Schematic of the building gene expression profile models for the healthy adult human brain. **(b)** Distribution of reproducible gene expression patterns across distinct brain structures for expression profile model, MD1. (**c)** Number of unique brain structures (BS) that pass or fail the gene expression pattern selection criteria for models, MD1 to MD4. (**d**) Brain structure based gene enrichment. Top ten brain structures with highest number of genes showing significant and differential expression across four models.

The same common set of principles was used to quantify differential gene expression (with 58,692 probes) patterns among different brain structures for each donor brain. For each gene in each donor, the gene expression data distribution across distinct brain structures was transformed into z-scores to identify differentially expressed gene patterns. Using z-score thresholds (−2.0 ≥ z-score ≥ 2.0) and unidirectional expression of genes (up or down) across six donors as consistent criteria, we developed four distinctive models (MD1-MD4) to find conserved patterns of transcription in precise anatomical structures. Each model differs in the number of donor brains and distinct brain structures that qualify with usable z-score data for model building. For the first model (MD1), usable data must exist in at least two-thirds of the donors and two-thirds of the identical anatomic brain structures. For the second model (MD2), all six donors and all brain structure must be representative of usable z-score data. In MD3, if the brain structures were sampled from four or less donor brains than the usable data must represent all the donor and all brain structures, however, if the brain structures were sampled in five or all the six donors than two-thirds of the structures suffice for finding conserved patterns. For our final model MD4, only the brain structures that were sampled in four or more donor brains were considered for finding conserved transcriptional patterns and at least two-thirds of these brain structures must have usable z-score data. As described, these criteria differ considerably in capturing the gene patterns (variant selection stringency) in identically annotated brain structures and this recapitulation of expression via distinctive criteria was crucial in establishing reliable expression profiles. The bar graph in Fig. 1d reports the top 10 brain structures that have the most number of differentially expressed genes among all the models. Brain structure based exhibit of gene expression established the blood brain barrier i.e., choroid plexus (CPLV)^27, 28^ as the transcriptionally most active brain structure followed closely by paraventricular nuclei and corpus callosum.

For our first model (MD1), we observed that about 85% (24,863) of the genes demonstrate synonymous expression profile in at least one brain structure. These results corroborate with the results from previous studies, which have shown 84% of genes to be expressed in the adult human brain^2^ and 80% in the mouse brain^26^. Comparison of expression patterns resulted in a total of 190 brain structures carrying similarly pattered gene expression for at least one gene. The frequency distribution of genes with reproducible expression patterns across distinct brain structures for MD1 is shown in Fig. 1b. Our results demonstrate the consistency of expression patterns across six donors. However, due to the stringency of filtering criteria, the frequency distribution of similarly expressed genes across brain structures is varied across different models. About 54% of the genes are expressed in at least one brain structure in MD2, 69% in MD3 and for our last model MD4, 59% of the genes were expressed in at least one brain structure. Since MD1 models shows the minimum loss of gene expression information by retaining the most number of brain structures with differentially expressed genes when compared to the other three models (Fig. 1c), we used the MD1 model for all the subsequent analyses in this study.

### Spatial organization of brain transcriptome

**Figure 2:**
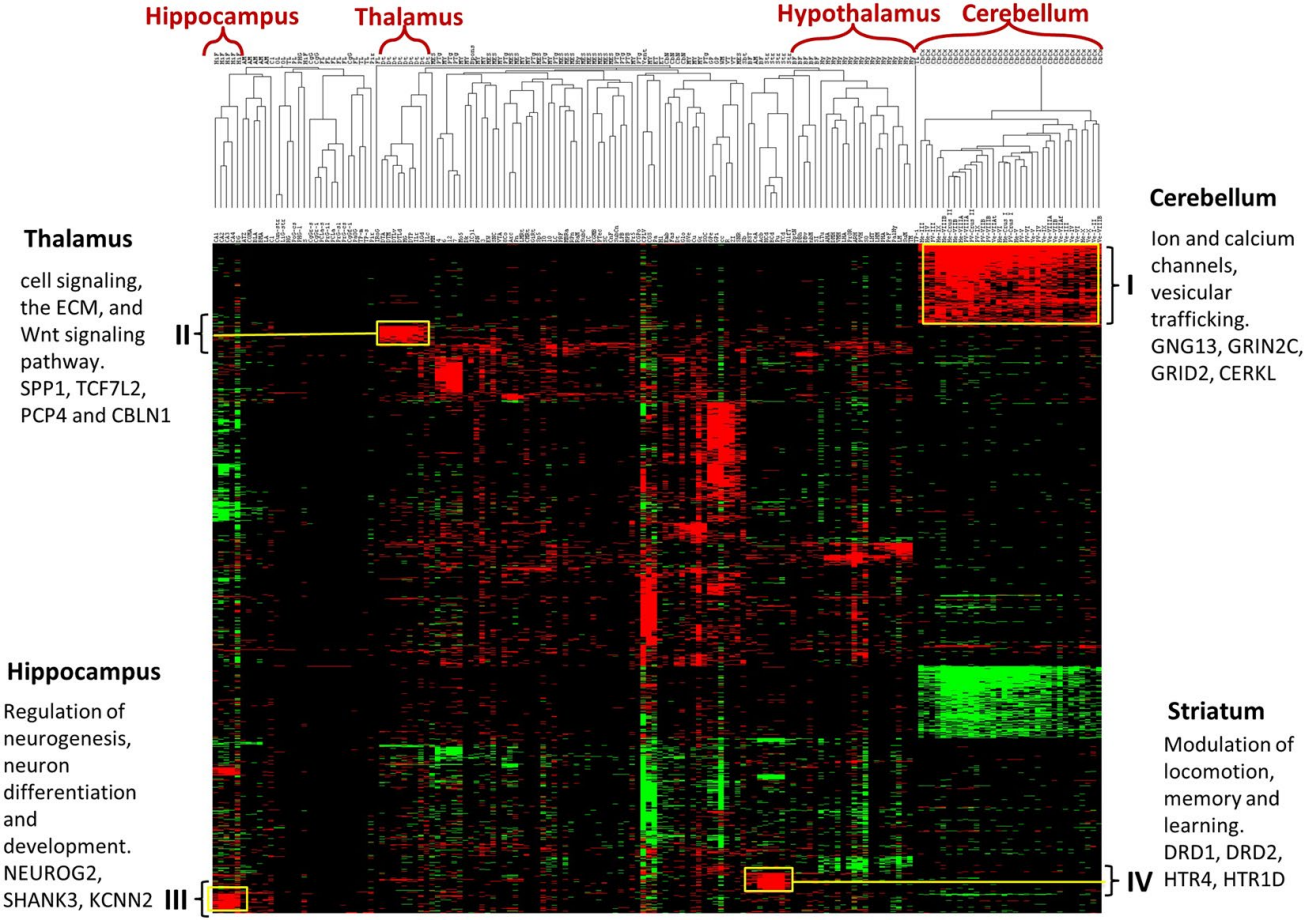
Dendrogram and heat-map overview of the two-way unsupervised hierarchical cluster analysis of gene expression data from 160 samples using 6,984 genes. Columns represent individual brain structures and rows represent each gene and the z-scores were calculated across rows. The expression level of each feature (gene) in every brain structures is represented as a cell in a two dimensional matrix. Red and green reflects up and down regulated expression levels, respectively, in a given brain structure with respect to all the other brain structures. Large transcriptional differences are seen between major structures. Cerebellum displays the minimum internal heterogeneity whereas there is a clear distinction in the expression patterns of cortical regions especially motor and sensory structures.

Hierarchical clustering^29^ of gene expression data provides a holistic view of the transcriptome organization in a full set of brain samples. To gain better insights into the structural and functional similarities between distinct anatomical locations, we checked for spatial grouping of 190 brain structures based on the expression of 6,984 genes showing significant differential expression in at least five brain structures (Fig. 2). Clustering using the full complement of expressed genes has been reported in the Supplementary Figure S2.

As shown in Fig. 2, using hierarchical clustering we found that the distinct structures of a bigger region can cluster together pointing towards underlying functional similarities. At a coarse level, the clustering patterns can be condensed into three groups. First, sets of genes expressed largely across the brain were identified, suggesting housekeeping functions. Second, we found cliques of genes in functionally related brain structures. Last but not the least, we also observed an array of genes that express only in a singular brain location, signifying their functional significance in discreet brain locations. By performing enrichment analyses on these gene groups, we identified biological processes and molecular functions, which are either specific to the brain’s functional and anatomical groups or represent generic cellular processes.

Most genes across brain regions are not selective for a single major brain region; rather, they are expressed in multiple regions in a non-uniform fashion. This suggests that a number of genes are pleiotropic to brain functionality^2^. Our data suggests that the cerebellum has the most distinguishable gene expression patterns of all the brain regions and displays the least internal heterogeneity^5, 30, 31^. Most of these genes when studied with enrichment analysis showed enrichment for ion channels as a dominant GO class for the upregulated gene cluster “I” (Fig. 2), which consists of 865 genes. It has been previously reported that the ion channels are enriched in cerebellum^32, 33^. Also, enrichment of genes associated with vesicular trafficking as well as E3-ubiquitin-protein ligases that play a role in DNA damage signaling was found in our results for cluster “I” and this result is in congruence with the existing knowledge^34^. Critical role of transmembrane ion flux via transporters and channels in various functions of cerebellum is also well established, including neuronal signal transmission and electrolyte homeostasis^35^, and we found that our results are in support of this concept. Some of the major gene players in the upregulated cerebellum cluster were GNG13, CERKL, GRIN2C, and GRID2. For example, the upregulation of GRIN2C in the adult cerebellum has been previously reported during the innervation of mosey fibers into granule cells^36, 37^.

About 740 genes were downregulated in cerebellum in the cluster labeled “II” (Fig. 2). We determined that the transcriptome of cerebellum as a whole possess a rich homogenous gene expression structure which might reflect the underlying cellular composition of the brain tissue. Other brain structures, which show conserved patterns, include hippocampus, amygdala, hypothalamus, dorsal thalamus, striatum and cerebellar nuclei. With the exception of the aforementioned brain structures, not many genes showed differential expression amongst the cortical regions such as occipital, parietal, frontal and temporal lobes and these results are agreeable with existing literature^5^. The hippocampal cluster labeled “III” (Fig. 2) showed significant over-representation of terms like neuron differentiation, neuron development, cell morphogenesis involved in neuron differentiation, regulation of neurogenesis etc. This is expected because hippocampus is the site for adult neurogenesis^38^. Genes such as NEUROG2, a transcriptional regulator involved in neuronal differentiation^38^; GRIA1 and SHANK3, genes known to be implicated in schizophrenia and autism^39, 40^; and KCNN2 were all part of the cluster “III”. Dorsal thalamus showed association with nicotine related GO categories such as nicotine acetylcholine gated receptor-channel complex and behavioral response to nicotine. Local clusters for striatum and its sub-divisions show high expression for dopamine receptors DRD1, DRD2, DRD3. Striatum is a brain structure where dopamine exerts its maximum effect as the dopamine producing neurons have their cell bodies in substantia nigra, which projects into the striatum^41^. High expression of HTR isoforms were also found in striatum. HTR and its isoforms are known to regulate the release of dopamine and regulation of extracellular dopamine, thereby affecting the neural activity^42^. Significant enrichment of PDE10A was found in the striatum sub-structures. An association has been established between the striatal expression of PDE10A gene and bipolar disorder patients^43^. The highly upregulated cluster in hypothalamus was enriched in molecular functions such as hormone activity and response to endogenous stimulus. Most prominent genes in this category were TRH, CRHBP, and GHRH. Other GO categories showing over-representation in hypothalamus included steroid hormone stimulus, neuropeptide hormone activity, response to corticosteroid stimulus, estrogen stimulus and response to glucocorticoid stimulus.

Taken together, the transcriptional profiles of the sub-divisions of bigger structural units are well conserved across six individuals, demonstrating transcriptional similarity amongst functionally related structures.

### Co-expression network construction

There are numerous ways to analyze multi-dimensional gene expression data; however, correlation networks provide a comprehensive outlook on the intrinsic organization of a transcriptome. Gene co-expression networks investigate gene-to-gene relationships in an unsupervised way and cluster coordinately expressed genes into modules. This provides a framework to better understand gene expression patterns in distinct brain structures, which may be driven by distinct cellular and biological processes. We used weighted gene coexpression network analysis (WGCNA) package in R to construct these networks.

**Figure 3:**
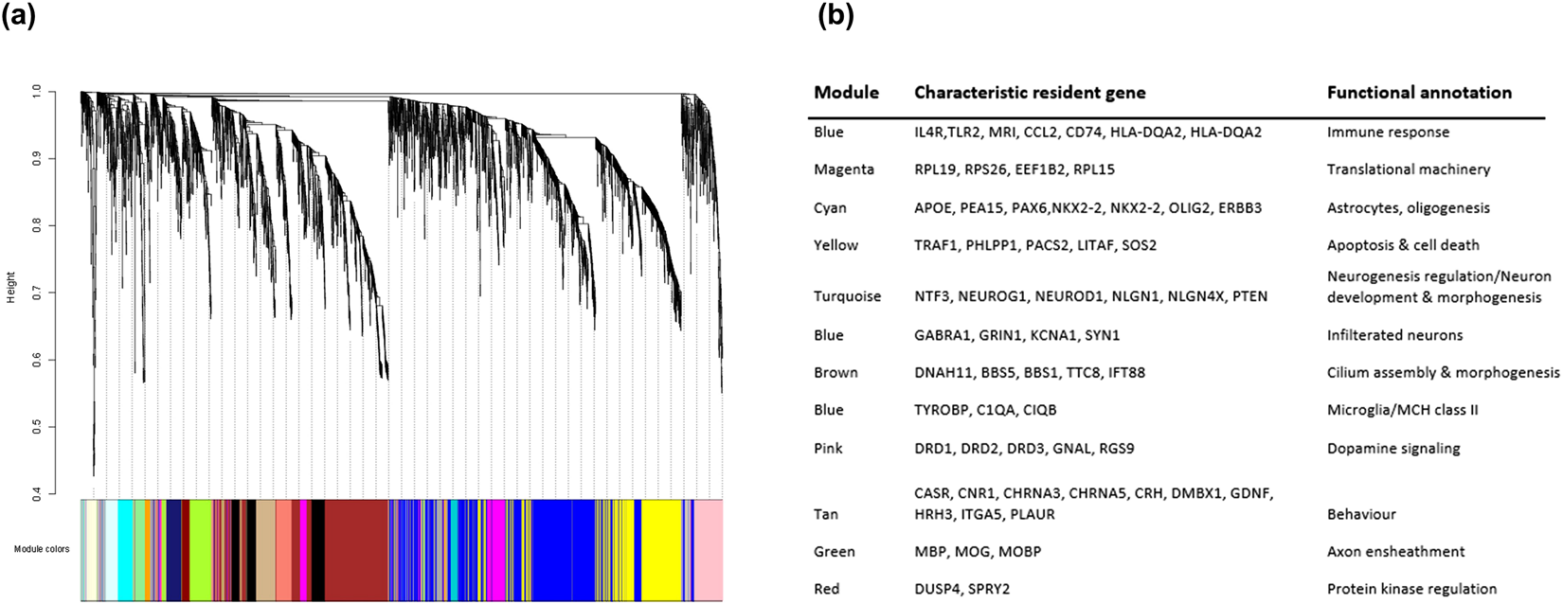
(**a**) Weighted gene coexpression network and gene modules. Dendogram depicting distinct colored modules of co-expressed genes. Using topological overlap as the dissimilarity measure and average linkage unsupervised hierarchical clustering of genes, 19 modules of co-expressed genes were identified by implementing dynamic branch cut to the dendogram. Each module was assigned a unique number (M1 to M19) and color. **(b)** Functional annotation of the modules: Genes that are characteristic of their module of residence have been carefully noted along with the top overrepresented functional (Biological process or molecular function) terms for each module.

Same set of 6,984 genes as previously discussed was used to construct the weighted gene coexpression network. Based on the TO (topological overlap), WGCNA evaluated the coexpression for every pair of genes, while simultaneously considering the degree of shared neighbors for every gene pair across the whole network. This results in the discovery of consistent gene coexpression patterns in the transcriptome. Our analysis resulted in 19 modules of highly co-expressed genes. Modules are groups of genes exhibiting high intra-module topological overlap. Each branch in the dendogram (Fig. 3a) is representative of a color-coded module containing a cluster of highly connected genes. Each module was assigned a unique color and the number of genes assigned to a module varies, ranging from 67 to 1469 (Supplementary Table S1). To further explore the co-expression relationships within a module, we summarized the first principal component for all the modules. Here, we obtained a single characteristic expression profile ‘ME’ (module eigengene) that is representative of the hub genes in each module. The resultant ME brings together the dominant expression patterns and helps to represent each module in a summarized form. Also, we quantified the connectivity measure for every gene within its resident module. We measured the module membership (kME) for each gene by correlating its gene expression profile with the ME of a given module. Genes participating towards high module membership or highly connected genes are referred to as hub genes and these genes represent major biological processes and molecular functions for the module.

### Biological relevance of network modules

The functional relevance of the gene module was assessed to make sure that the modules designed by co-expressed genes convey biologically relevant information. Gene ontology enrichment analysis was performed to examine the ontology terms over-represented in the modules. Also, KEGG orthology based enrichment analysis was performed using hypergeometric tests for each module. A wide range of functional association was configured with the statistical analyses of gene composition, which can be grouped into several categories as summarized in Fig. 3b.

Notably, a significant over-representation of the dopamine receptor-signaling pathway, G-protein coupled receptor signaling pathway and catecholamine binding was observed for the pink module consisting of 225 genes. Structurally, Amygdala and striatum sub-structures are the predominant regions accommodating the pink module genes. Role of dopamine in amygdala has been well studied specially with regards to D1 and D2 receptors^41, 44^. Yellow module was enriched for biological processes like cell death, regulation of apoptosis and regulation of programmed cell death. Top genes for the yellow module included TRAF1, PHLPP1, PACS2, LITAF, PREX1 and SOS2. Upon comparison with results from published literature^2, 3^, we found that the module enrichment results in our analysis have provided novel findings that have not been previously reported (Supplementary Table S1). Also, the yellow module in our study is enriched for chemokine signaling pathway and neurotrophin signaling pathway, suggesting reduced neuronal support to maintain homeostasis in the adult human body^45^. On similar lines, enrichment for cilium organization, morphogenesis and cilium assembly was found in our co-expression analysis, which were not reported in any of the previously published large scale co-expression analyses using the same data. The top 200 genes from the brown module with the highest K-within (intramodular connectivity) showed significant enrichment of primary cilium and cilium assembly for neuronal functions. The major gene players in the brown module included DNAH11, BBS5, BBS1, TTC8 and IFT88^46, 47^. Biological processes like neuron development & morphogenesis and regulation of neurogenesis were significantly enriched in the turquoise module, which also happens to be the largest with 1,469 genes. Genes like NEUROG1, NTF3, NEUROD1, and NLGN1 were the highly-connected nodes in this module. Turquoise module genes could be mapped back predominantly to cerebellum and hippocampus. Blue module was most strongly enriched for GO terms such as immune response, microglia and MCH class II.

### Neuroanatomically indigenous functional annotation

Since larger global transcriptomic signatures can mask signatures of local phenomena, we performed local analysis on smaller brain structures of a bigger anatomical region. Here, we show in-depth biologically enriched similarities/differences between the sub-structures of hippocampus.

**Figure 4:**
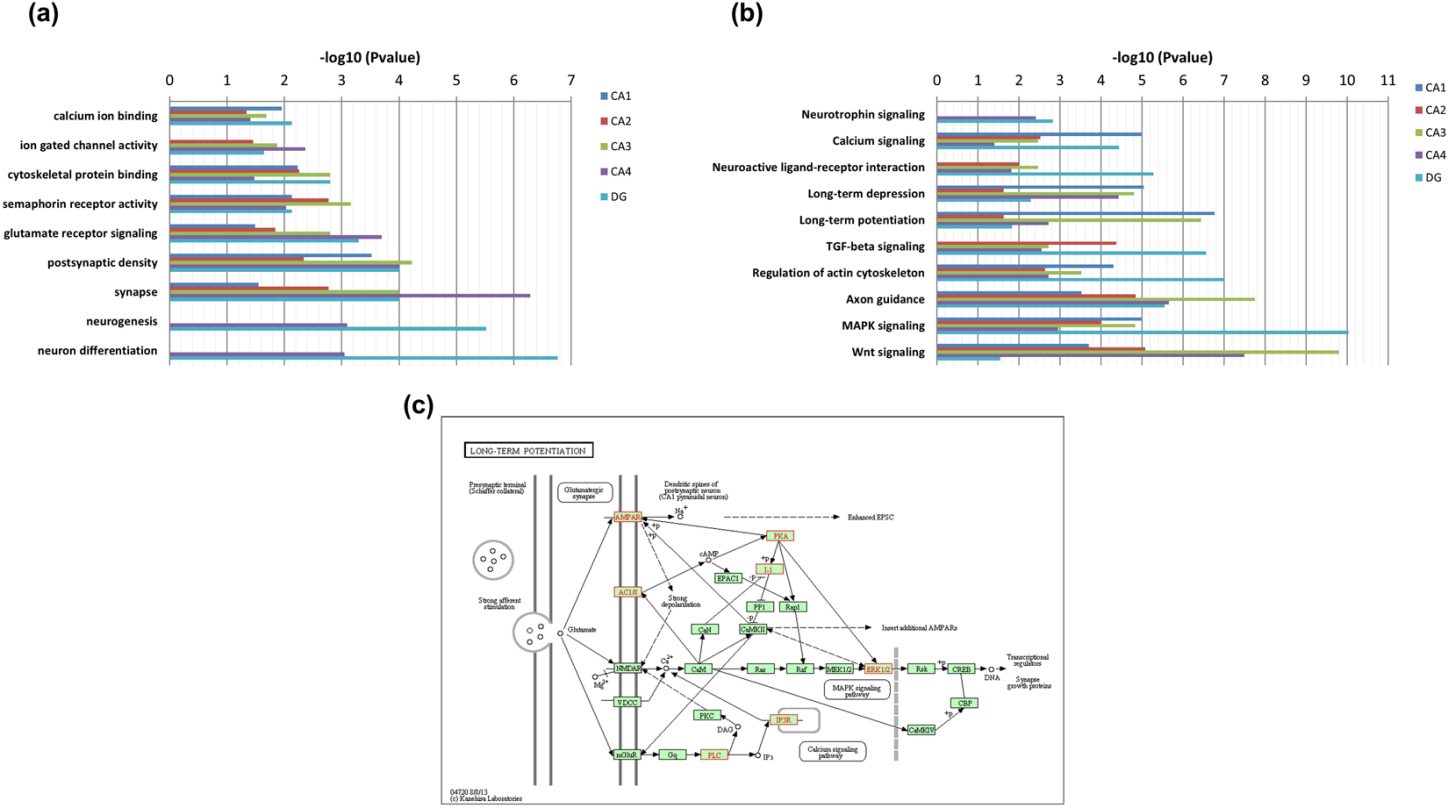
Bar graphs summarizing the pathway and GO enrichment analysis for the different substructures of hippocampus. **(a)** Top 10 significant GO terms associated with the genes expressed in each of the hippocampal substructures. The horizontal axis represents the –log10 (p-value) for the GO terms represented on the vertical axis. Enrichment of terms related to neurogenesis and neuronal differentiation was found specific to dentate gyrus (DG) and cornus ammonis (CA4) regions, while synapse-associated genes are particularly enriched in CA4 region. **(b)** KEGG pathway enrichment had neurotrophin signaling, neuroactive ligand-receptor interaction and long term potentiation as the top enriched brain tissue-specific pathways. This pathway image is obtained from KEGG^77, 78^. **(c)** Long-term potentiation pathway mapped with the genes enriched in DG is known for its role in neurogenesis and other hippocampus specific functions. The genes of interest have been highlighted in red color.

Gene ontology (Fig. 4a) and pathway (Fig. 4b) enrichment was performed on each hippocampus sub-fields (dentate gyrus, CA fields and subiculum) using the significantly differentially expressed genes. One of the notable findings was neurogenesis as the top biological process for dentate gyrus (DG) and cornus ammonis (CA4) subfields. It has been previously established that adult hippocampus is the sight of neurogenesis^48^; essentially DG and CA4. Furthermore, long-term potentiation (LTP) enhances the neurogenesis process in DG^49^, and interestingly LTP was the leading pathway in our results. Upon inspecting the genes enriched in DG and correspondingly in LTP, we found co-occurrence of AMPAR, Plcb1, Mapk1/3, Ascl1, Adcy1 and IP3 in both DG and LTP. We mapped these genes on the LTP pathway as shown in Fig. 4c. AMPAR is known for its role in postnatal hippocampal neurogenesis^50^. During LTP, dendritic NMDA and AMPAR receptors are involved in the development of new synapses. Similarly, MAPK1/3 and Plcb1 have been documented^51^ as genes regulating adult hippocampal neurogenesis and neuronal differentiation. Also, Ascl1 has been widely recognized to regulate gene expression during neurogenesis and neuronal differentiation^52^ and Adcy sub-types have been connected with memory processes. Similarly, robust regional patterns of biological importance were observed in striatum, globus pallidus, amygdala and dorsal thalamus (Supplementary Figure S3a, S3b, S3c and S3d).

### Neurotransmitter system maps

Neurotransmitters play a very important role in the overall machinery of brain function. Mapping the structural distribution of the major neurotransmitter receptors can provide novel and functionally more relevant insights into the spatial organization of the human brain. We mapped the pathway distribution of five major neurotransmitter systems- serotonin, dopamine, choline, GABA and glutamate.

**Figure 5:**
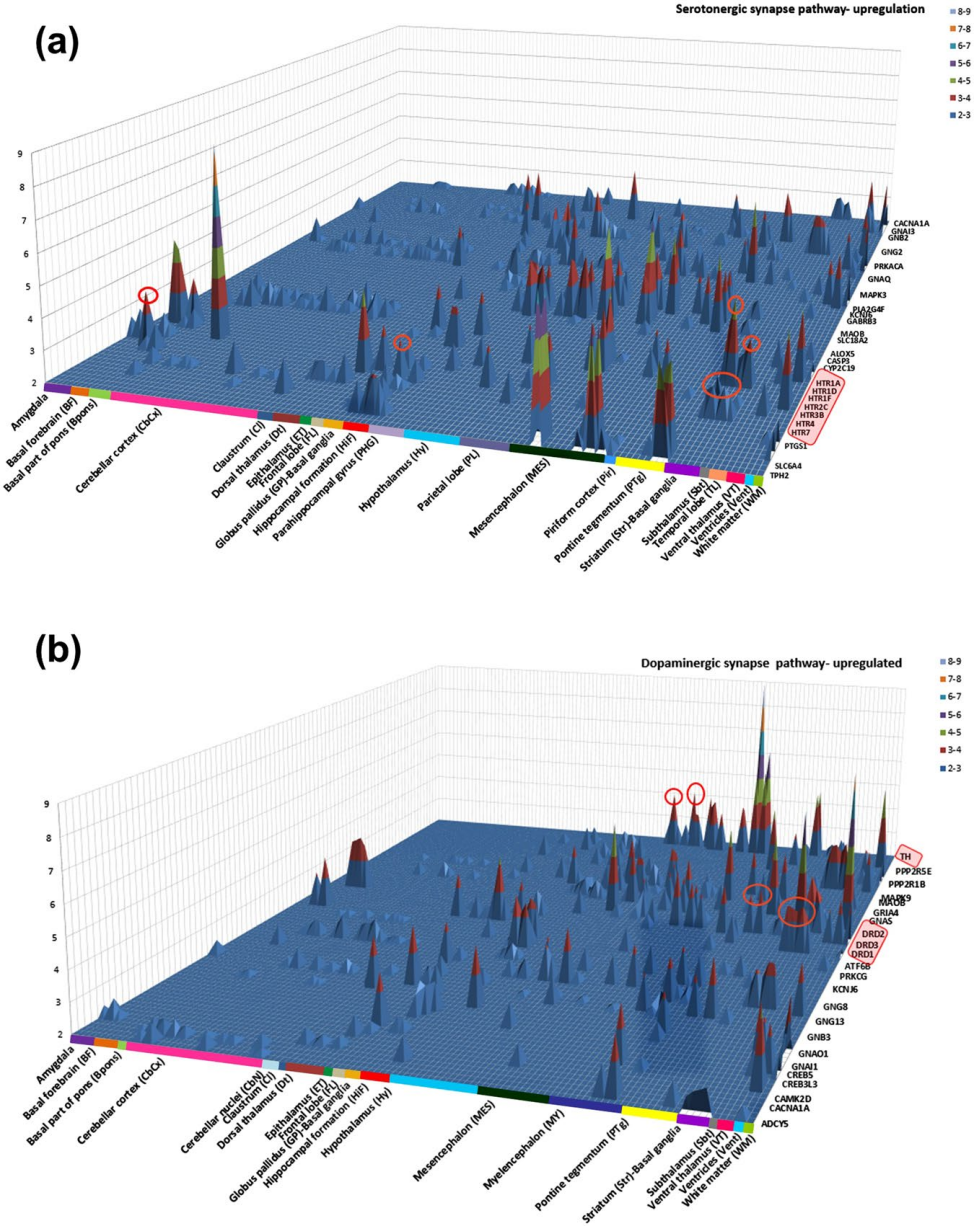
Structural distribution of gene expression in neurotransmitter systems. Distribution patterns of receptors shed light on the relation between anatomical units and their functions. **(a)** Serotonergic synapse showed spatial enrichment of HTR receptors in substantia nigra (mesencephalon) and globus pallidus (basal ganglia). **(b)** Dopaminergic synapse had significant enrichment of DRD receptors in striatum and amygdala.

Serotonin (5-Hydroxytryptamine, 5-HT) is a monoamine neurotransmitter playing an important role in physiological functions such as learning and memory, pain, endocrine secretion, as well as states of abnormal mood and bad cognition^53^. The serotonin 5-HT6 receptors are located primarily in the striatum^54^, and the mapping of receptors in our study shows enrichment patterns in support of this observation. We also found other HT receptors like HTR7, HTR4, HTR1D and HTR1A to be significantly present in striatum. Another interesting finding was the enrichment of HTR1A and HTR1B in substantia nigra (mesencephalon), hippocampus and hypothalamus. Also, a high expression of HTR1A receptor was observed in cerebellum. The detailed map of serotonin synapse participating molecules can be seen in Fig. 5a.

For the dopaminergic synapse, a noticeable enrichment of tyrosine hydroxylase in substantia nigra pars compacta (a brain structure located in mesencephalon) was observed as shown in Fig. 5b. Dopamine serves as a precursor for noradrenaline for the neurons in these locations^2^. Also, high expression of DRD1, DRD2 and DRD3 was seen in striatum as expected^55^. DRD2 was also enriched in substantia nigra (SNC), ventral tegmental area (VTA) and hypothalamus. Since, VTA is the origin of the dopaminergic cell bodies of the dopamine system significant expression of DRD receptors is expected to be high^56^. Detailed maps of other neurotransmitter pathways are included in Supplementary Figure S4c, S4b, S4c.

### Prediction model using healthy brain gene expression patterns

Evaluating the utility of healthy brain gene expression as a tool to predict potentially new genes for a neurological disorder offers a massive scope in incrementing our current knowledge. In the present study, we used the discriminatory power of the expression patterns of known autism-associated genes in a healthy individual and developed a model to predict new genes with potential association to autism. Based on random forest an overall class prediction accuracy of 84% was achieved. The sensitivity and specificity was 0.84 and 0.60 respectively.

**Figure 6:**
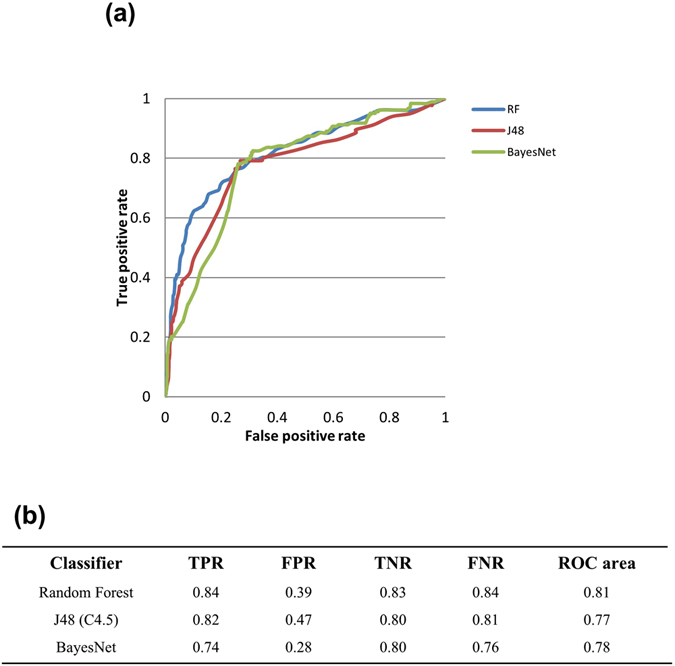
(**a**) ROC curves showing the relationship between TPR (sensitivity) and FPR (1-specificity) for the three ML algorithms used for model building. Most optimal results were obtained by RF with an accuracy of 84% and the AUC of 0.81. **(b)** 10-fold cross-validation performance measures of three classifiers.

For the prediction model building, 219 autism implicated genes from the AutDB^57^ database constituted the positive dataset. Their expression profiles across the 190 brain structures were extracted from our healthy brain expression model and served as the feature vectors. For the negative dataset, 830 brain-enriched genes were selected from the Protein Atlas^58^. Similarly, their expression profiles were extracted from the healthy brain expression model. Note that both the positive and the negative training datasets come from the gene expression profiles of the brain. Using these datasets, we labeled each gene with its assigned class (autism-associated and non-autism associated) and developed a classification model to predict the classes of unseen or novel autism associated genes. Three popular machine learning (ML) algorithms were tested; Random Forest (RF)^59^, BayesNet^60^ and J48^61^, to find the most appropriate algorithm for our dataset. Random division of all the feature vectors was conducted to generate 80 and 20 percent subsets for training and testing, respectively. Since the datasets are unbalanced across classes, using stratified partitioning we preserved the approximate class distributions for training and testing sets. A two-step validation technique was used. In step one; we determined 10-fold cross-validation accuracy on the training set. For step two; using the testing dataset that is not a part of the training data we determined the testing accuracy of the model. We also report standard performance measures of each class, including true positive rate (TPR), false positive rate (FPR), and receiver operating characteristic (ROC) curves and the area under the curve (AUC). Figure 6b shows the performance measures of each ML algorithm. Out of the aforementioned ML algorithms, we selected RF method for further use in this study owing to its superior performance. Also, J48 algorithm achieved a close accuracy with a slight loss in TPR and FPR measure. Figure 6a illustrates the ROC curves showing the relationship between TPR (sensitivity) and FPR (1-specificity). In an ideal scenario, ROC curve goes straight up on the Y-axis and then to the right parallel to the X-axis; thereby maximizing the AUC. An AUC close to 1 indicates that the classifier is predicting with maximum TP and minimum FP. We calculated an AUC of 0.81, indicating that the classification model can markedly differentiate between the autism versus non-autism associated genes. We have also demonstrated the use of this generic methodology and provided an additional prediction model to predict genes associated with Parkinson’s disease. We used three different machine learning algorithms; RF, J48 and BayesNet and achieved the best accuracy of 81%. The results on Parkinson’s disease genes can be found in the Supplementary Figure S6.

## Discussion

In this study, we have developed a new framework to systematically integrate high throughput transcriptome profiling data from healthy human brains, complemented with various functional analysis techniques for the generation of a gene functional map. The brain structure specific gene expression profile generated from this framework served as a baseline reference for the development of a generic prediction model to find new disease implicated genes for any given neurological disorder.

Our study was designed on three objectives. First, we wanted to reduce the complex brain gene expression into recurring patterns where the spatial information is conserved. Since reproducible gene expression patterns across neuroanatomical structures in different individuals tend to have properties fundamental to brain’s functioning^2, 3^, defining these brain specific and general transcriptional patterns is essential. In our second objective, we wanted to functionally characterize the transcriptional landscape of the human brain as captured in our gene expression model. Implementation of clustering, network analysis and receptor mapping suggested striking features of global and local transcription patterns. The third impetus for this study comes from the paucity of brain expression data to study neurological conditions. So, in this study we were also able to move towards developing a prediction model, which would be effective to identify new genes with potential association to a neurological disorder, using the reproducible gene set of healthy brain gene expression. As an example, we have demonstrated the utility of this method to predict genes that might be involved in autism and evaluated the performance measures of the classifier. This, to the best of our knowledge, has never been presented before.

While building the expression model we noticed high recurrence of similar expression profiles across the similarly annotated samples from six individuals suggesting high recapitulation of the brain’s transcriptome^1–3, 26^. Genes in the adult human brain show significant spatial heterogeneity, however, discrete patterns within higher-level anatomic structures were also observed (Fig. 2). Groups of genes based on similarity in gene expression profile were found that would ‘occur naturally’ and can point towards putative co-regulated genes. We found that there are some such coherent gene groups (cerebellum, hippocampus, striatum, dorsal thalamus, amygdala), but many, of the genes exhibit high variation over different brain structures and do not lie in any of these groups. Additionally, mapping of the structural distribution of neurotransmitter systems revealed interesting patterns of enrichment in localized regions for various receptors. We found a high expression of HTR2B in cerebellum in the serotonergic neurotransmitter system which is in contrast to its detection at lower expression levels in cerebellum along with occipital and frontal cortex as previously reported^62–65^. These structural distribution maps provide an important overview of anatomical, functional and molecular organization of these receptors.

We also performed an exhaustive assessment of co-expression patterns in the human brain expression map. For example, the pink module, which showed properties related to dopaminergic synaptic function (Fig. 3b) was identified in striatum and amygdala. This finding is consistent with the predominant neuronal phenotype made of medium spiny neurons that have been associated with high expression of DRD receptors^66^. Most modules appeared to display a particular biological function: the magenta module reflects the translational machinery with resident genes such as RPL19, RPS26, EEF1B2f, and the cyan module reflects glial cell differentiation/oligodendrocyte differentiation and astrocytes enriched with genes like NKX2-2, NKX6-2, OLIG2, APOE, PEA15 and PAX6. Functional enrichment of genes in each of the coexpression modules thus reflects the molecular events in the human brain.

Finally, we used machine-learning classifier that learns the expression patterns of known autism spectrum disorder (ASD) genes in the distinct brain structures and then uses the expression driven indicators specific to ASD genes to predict novel genes from the full complement of the genome. We explored the predictive capabilities of 219 autism genes in the framework of healthy brain expression data. The classification performance based on our model is encouraging, especially given the use of healthy brain data to make predictions.

A major strength of our study is the utilization of the Allen Human Brain Atlas to design the blueprint gene expression model in representing a healthy state. Conceptualized on the ‘all gene- all structure’ approach, ABA allows to explore the transcriptome properties at the deepest structural anatomy of the human brain. Also, this study is focused on those genes alone that exhibit compelling experimental evidence to autism susceptibility rather than the complete gene set catalogued in AutDB. There are also some pitfalls in our study. First, the sample size is relatively moderate constituting five male and one female donor. Also, sex biased transcriptional variation may be detrimental to our method^67^. However, since we are trying to capture the common core components of gene expression population wide, sex as a confounding factor can be disregarded^2, 3^. Second, we implemented strict expression pattern selection parameters where our aim was to capture genuine brain specific as well as general transcription patterns. In course, we anticipate losing some expression information. Third, the brain structure specific profiles have been established at RNA level and these may not translate at protein levels^68^. Finally, for a ML algorithm to perform efficiently the ratio of positive to negative training instances is crucial^69^. For this reason, we chose BayesNet (a modified Naïve Bayes) and Random Forest MLs since they are least sensitive to changes in the number of instances in the training set^70^. Nevertheless, the accuracy we have obtained in the current study is an important step towards predicting potentially disease implicated genes using healthy tissue.

In conclusion, this study describes conserved transcriptional profiles across distinct brain structures among healthy population. Application of diverse functional analysis methods on these homogenous gene expression patterns allowed us to refine the biological processes and pathways that are specific to brain structures, and provided a systems-level view of gene expression variation in the brain. Finally, to the best of our knowledge, this study demonstrates the first comprehensive utilization of healthy brain tissue gene expression profiling to predict disease-implicated genes in several neurological disorders.

## Methods

### Microarray Data from Allen Brain Atlas

Human microarray data from the Allen Brain Atlas was used to generate the healthy gene expression model. The data is comprised of six neurotypical adult individuals. The profiles of individual donors and the statistics on the brain structures are shown in Supplementary Figure S1. For each donor, the microarray datasets contain approximately 400–500 tissue samples per hemisphere, often with multiple samples per structure for each brain. Throughout the process, anatomic data (MRI, blockface images, histology) were annotated to enable the collection of anatomically defined samples for microarray. Also, due to normal variation in brain size and morphology, the number of samples per structure varies across brains. For two brains (H0351.2001 and H0351.2002) the samples were collected from both hemispheres. Otherwise, samples for microarray were collected from the left brain hemisphere alone. Therefore, to eliminate bias arising due to hemispheric specificity of gene expression, only left hemisphere of the donor brains were used in our methodology.

### Development of gene expression model

Since multiple samples per brain structure can be present, we merged the samples, which are identically annotated according to the anatomical information and averaged the expression values of all genes (Supplementary Figure S5). To estimate the expression profile of each gene in every distinct brain structure w.r.t other structures we calculated the z-scores. Statistically, the standard score or z-score is the signed number of standard deviations by which an observation deviates from the mean. We compute the z-score for each probe independently over all brain structures for all donors. In our method, each z-score for every gene represents the individual regional gene expression normalized to the whole brain expression of that gene. In this manner, each gene has a unique z-score for each individual brain structure. We used a cut-off of ‘−2.0 ≥ z-score ≥ 2.0’ to retain only the significantly differentially expressed genes in the matrix for each of the six brains independently. Cells in the matrix with missing values (that do not meet the z-score cutoff) were populated with ‘zeroes’. Subsequently, we designed four different criteria to select reproducible gene expression patterns across six whole brain gene expression matrices. In the microarray data, each gene is assayed with multiple probes (58,692 probes covering 29,165 genes). The probe that showed maximum variance across brain regions was selected to be the representative probe for each gene. The general schematic of the expression model is summarized in the flowchart in Fig. 1a.

### Hierarchical clustering

Hierarchical clustering of all the 191 samples (brain structures) was performed using uncentered correlation as the similarity measure and average linkage as the clustering method. Two-way clustering was used, where both the genes and the samples were clustered. Since, clustering the full complement of genes diminishes the significance of brain-specific gene expression; a cut-off was applied to the gene set to focus on the genes that are commonly expressed in brain. Here, a gene had to be significantly differentially expressed in at least five brain structures to be included in the gene set. This set of genes was also typically associated with highest expression variance. This reduced the number of genes to 6,984 and also the sample size of distinct brain structures was reduced to 166. Software implementation of the algorithm obtained from ‘http://bonsai.hgc.jp/~mdehoon/software/cluster/’ was used for clustering and the clustered files were visualized using ‘TreeView’ software^71^. Clusters of coordinately expressed genes were functionally characterized using ‘DAVID’ software^72^. The results were validated using ‘WebGestalt’^73^.

### Weighted gene coexpression network analysis (WGCNA)

WGCNA^74^ was performed in the R and a coexpression network was constructed on the basis of 6,984 genes. For all possible pairs of the variable genes, Pearson correlation coefficients were calculated across all samples. The correlations matrix was raised to the power 9, thus producing a weighted network. The weighted network was transformed into a network of topological overlap (TO)—an advanced coexpression measure that considers ‘not only the correlation of 2 genes with each other, but also the extent of their shared correlations across the weighted network. Genes were hierarchically clustered on the basis of their TO. Modules were identified on the dendrogram using the Dynamic Tree Cut algorithm. For each gene, we determined its connectivity within its module of residence by summing up the TOs of the gene with all the other genes in the module. By definition, highly connected (hub) genes display expression profiles highly characteristic for their module of residence. To obtain a condensed representative expression profile of each module (ME), we summarized expression levels of the top hub genes in the module.

Functional annotation of the modules was performed on the basis of analysis of their gene composition. Again, we used DAVID (http://david.abcc.ncifcrf.gov/) to test each module for enrichment in genes with particular GO terms and biological pathways compared with the background list of all genes on the array. The functional characterization of the modules was also validated using ‘WebGestalt’.

### KEGG database (for neurotransmitter maps)

We used Kyoto Encyclopedia of Genes and Genomes (KEGG), as reference pathways to map the neurotransmitter systems in the human brain. KEGG pathway database is a collection of manually drawn pathway maps of molecular interactions. Currently, it represents the most comprehensive database source integrating the genomic, proteomic and systemic functional information for pathway analysis^75^.

### Machine learning methods

We used the Waikato Environment for Knowledge Analysis (WEKA) 3.6.14 package^76^; an open-source, Java-based framework to build classification models using different ML techniques. Three most popular machine learning classifiers namely; BayesNet^60^, RF^59^ and J48^61^ classifiers were used to build gene classification models.

### AutDB dataset

AutDB^57^ is a publicly available web-portal for on-going collection, manual annotation and visualization of genes linked to autism. We downloaded all the 845 genes associated with ASD, including both rare mutations and common variants from the AutDB database. Genes having a predisposition to autism in the context of a syndromic disorder and genes demonstrating strong evidence for replication in an independent experiment after a rigorous statistical comparison between cases and controls were retained. All the other genes with minimal evidence and hypothesized but untested evaluations were filtered out. Finally, we had a list of 219 autism implicated genes with high confidence.

### The Human Protein Atlas

The Human Protein Atlas^58^ provides expression for all protein-coding genes in all major tissues and organs in the human body including the brain. A total of 1223 genes were shown to demonstrate elevated expression in brain compared to other tissue types in the Human Protein Atlas. Of these 1223 genes we filtered out any overlapping genes in the autism dataset derived from AutDB. Also, the ‘group enriched’ genes (genes which shown at least five-fold higher mRNA levels in a group of 2–7 tissues) as defined by the protein Atlas were filtered out, leaving us with a set of 830 genes with elevated expression in the human brain.

### Performance measurements

We used standard evaluation metrics that include 10-fold cross validation and ROC curves. In 10-fold cross-validation, genes were divided into ten parts and the class ratio was kept constant in each part. Models were trained using the other nine parts and the predictions were generated and evaluated on the data contained in part one. This procedure was repeated ten times, each part is tested against the models built using other nine parts. The average of performance measurements of all ten iterations is considered as an unbiased estimate of the whole classification model. After cross-validation, we assessed the performance of the fully trained classifier models using the test set (20% of original data) that were hidden from the classifiers. We report performance of the classifiers using evaluation metrics, including: accuracy, sensitivity (TPR, also called recall), specificity (true negative rate, TNR), false positive rate, false negative rate, precision (Positive Predictive Value, PPV) and F measure (also called F1 score). In addition, we generate ROC curves, which graphically present the performance of classifiers for each class and calculate the AUC as a numeric evaluation of ROC curves.

## Electronic supplementary material

Supplementary Figures

Supplementary dataset
