# Supplementary material for: Global gene expression profiling of healthy human brain and its application in studying neurological disorders
Source: Sci Rep. 2017 Apr 18;7:897. doi: 10.1038/s41598-017-00952-9 (PMC5429860; doi:10.1038/s41598-017-00952-9)
Supplement: Supplementary file 1 — Supplementary Figures [file 41598_2017_952_MOESM1_ESM.doc]

**An expression model of healthy human brain and its application in neurological disorders**

**Simarjeet K. Negi^1^ and Chittibabu Guda^1, 2, 3, 4*^**

**
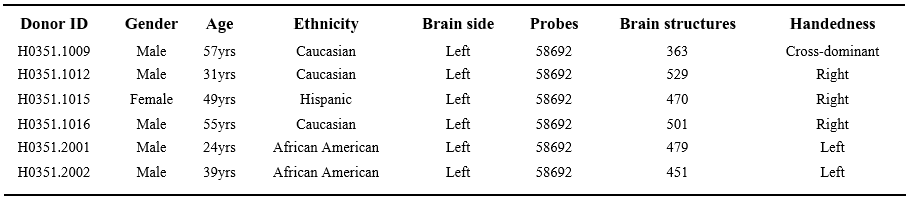
**

**Supplementary Figure S1.** The profiles of these six donors representing their age, gender, ethnicity, handedness and the number of samples obtained per left hemisphere

**
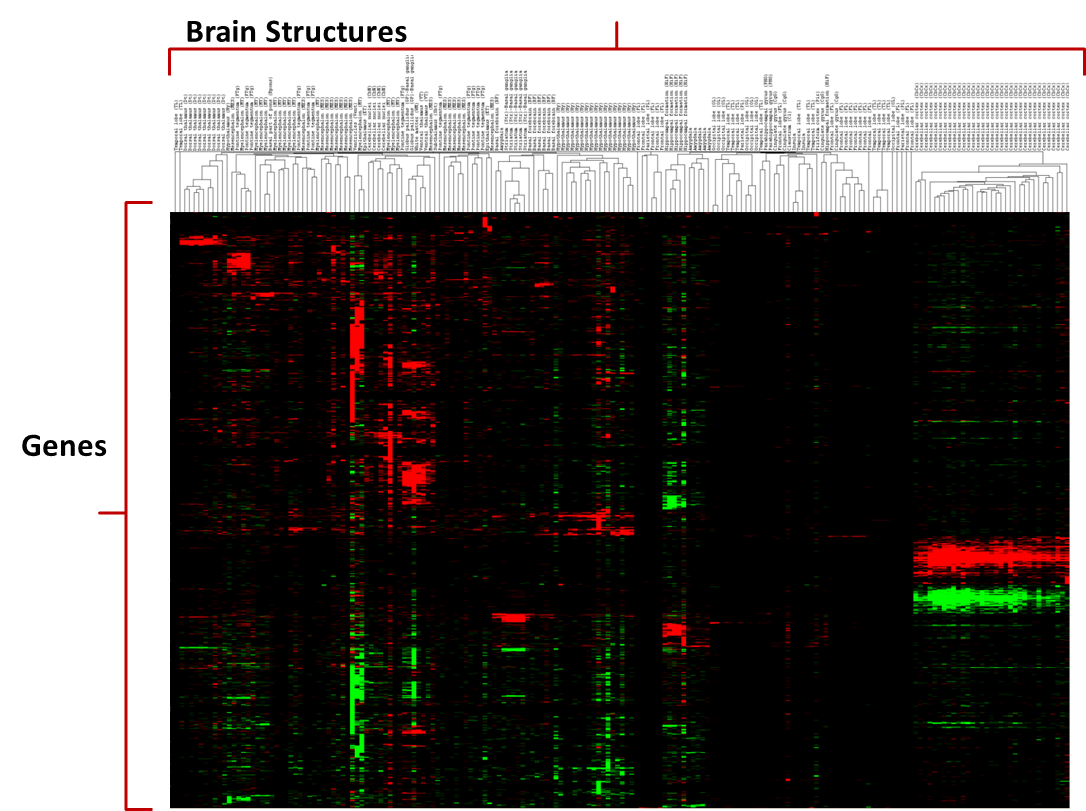
**

**Supplementary Figure S2.** Clustering using the full complement of expressed genes. Columns represent individual brain structures and rows represent each gene and the z-scores were calculated across rows. The expression level of each feature (gene) in every brain structures is represented as a cell in a two dimensional matrix. Red and green reflects up and down regulated expression levels, respectively, in a given brain structure with respect to all the other brain structures.

**Supplementary Figure S3a**
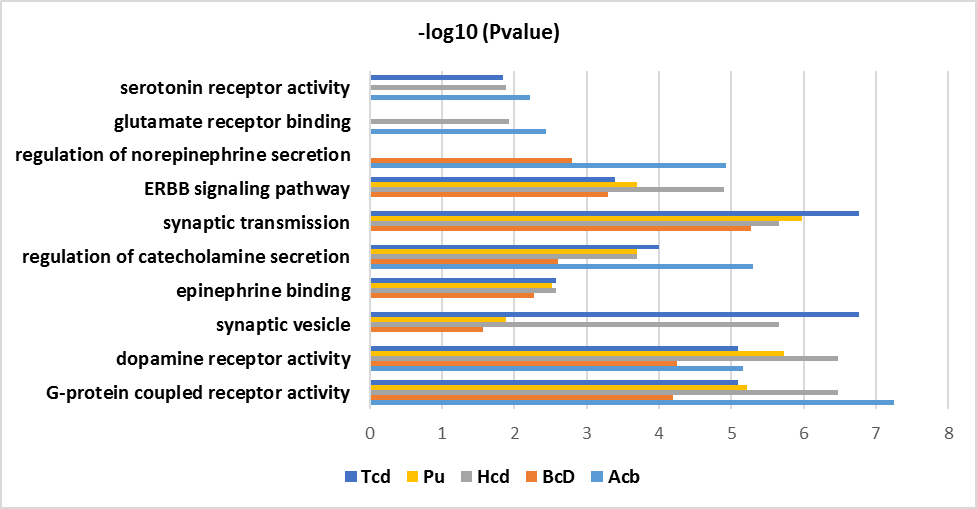
**.** Gene ontology enrichment on each of the striatum sub-fields. A significant over-representation of dopamine receptor activity is seen in the bar graph plot in figure S3a. This can be attributed to the fact that striatum is the brain structure where dopamine exerts its maximum effect

**
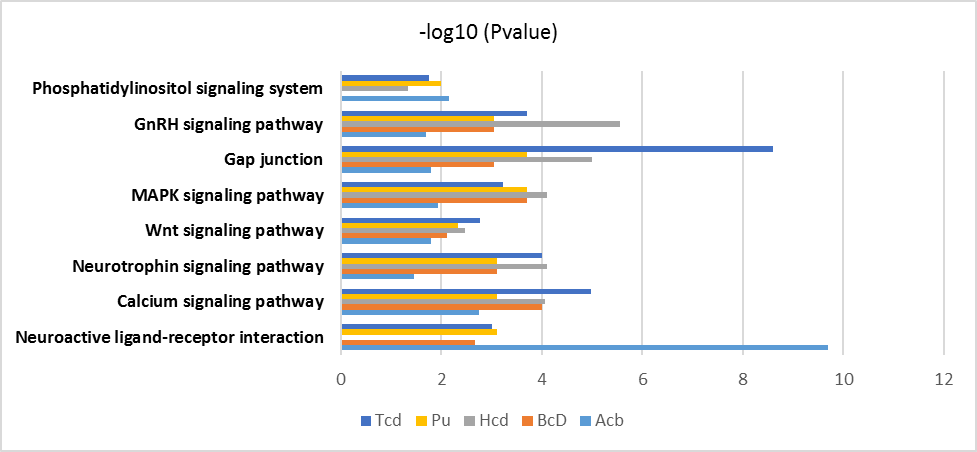
**

**Supplementary Figure S3b.** Pathway enrichment on each of the striatum sub-fields.

**
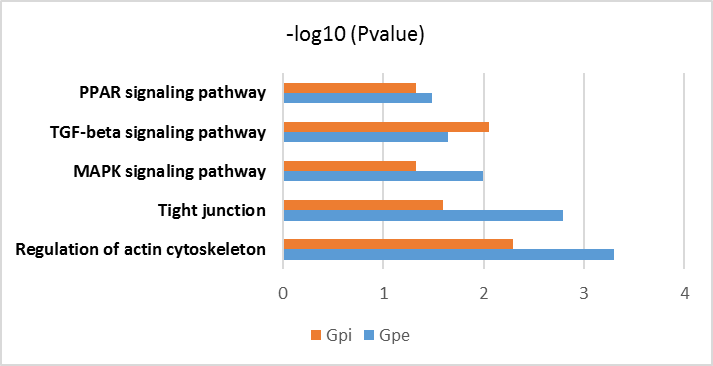
**

**Supplementary Figure S3c.** Pathway enrichment on the two globus pallidus sub-fields

**
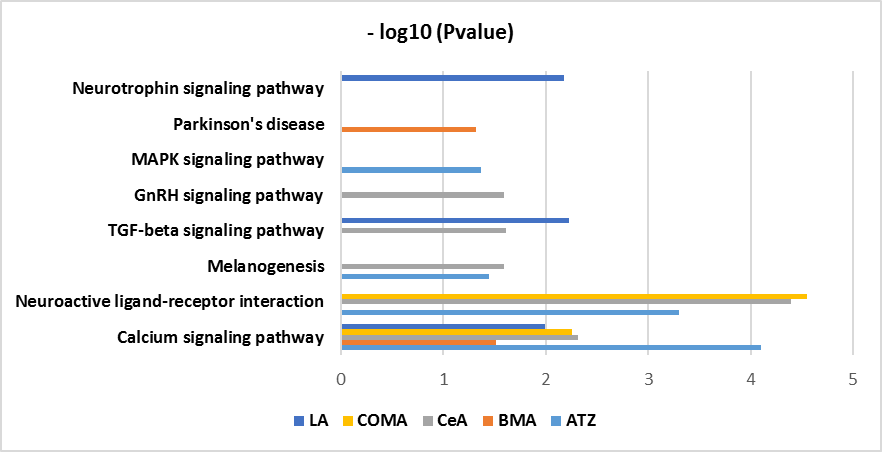
**

**Supplementary Figure S3d.** Pathway enrichment on the amygdala sub-fields. The pathways containing the highest numbers of affected genes are listed that contains neural-pathways such as Neuroactive Ligand-Receptor Interaction pathway, gonadotropin releasing hormone (GnRH) signaling, melanogenesis, neurotrophin signaling. Since amygdala shows pathological changes in Parkinson’s disease, it was one of the over-represented pathways for the basomedial amygdala (BMA) in our data

**
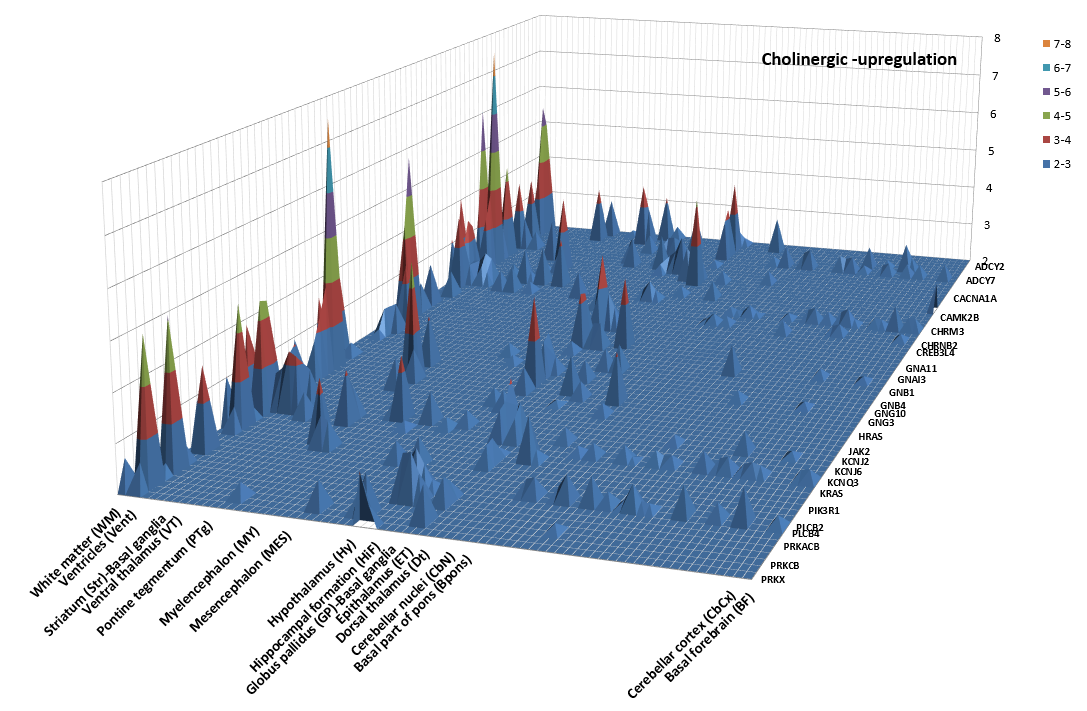
**

**Supplementary Figure S4a.** Structural distribution of gene expression in the cholinergic neurotransmitter systems

**
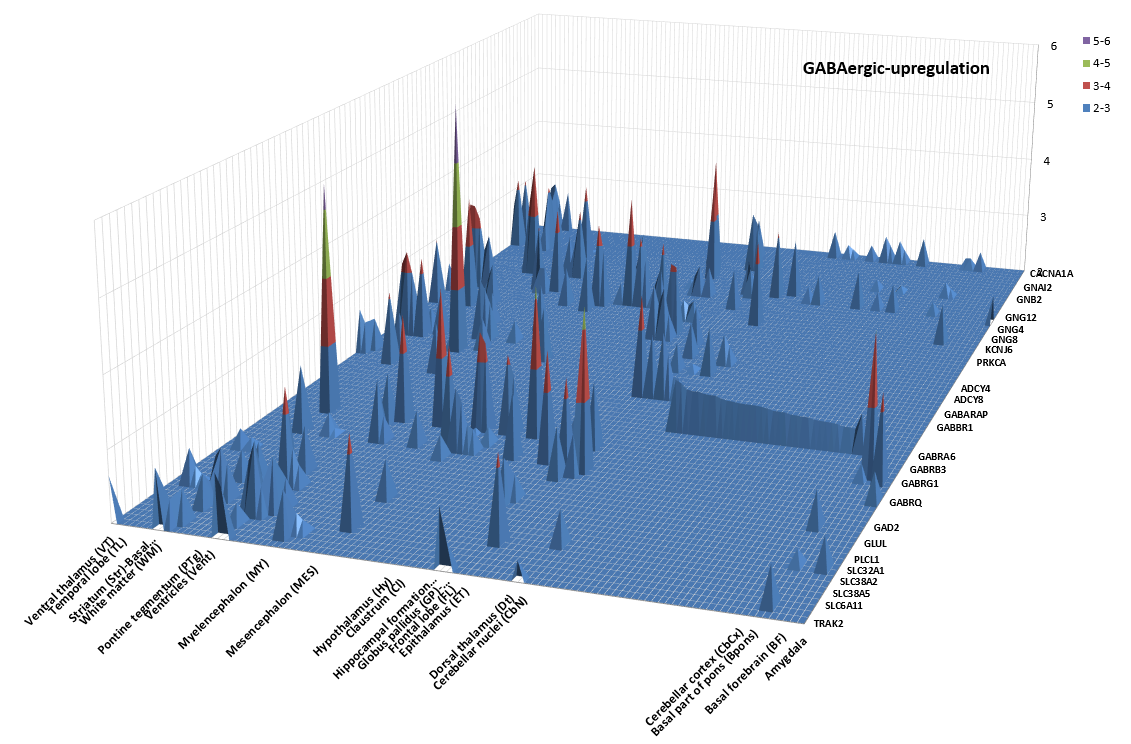
**

**Supplementary Figure S4b.** Structural distribution of gene expression in the GABAergic neurotransmitter systems

**
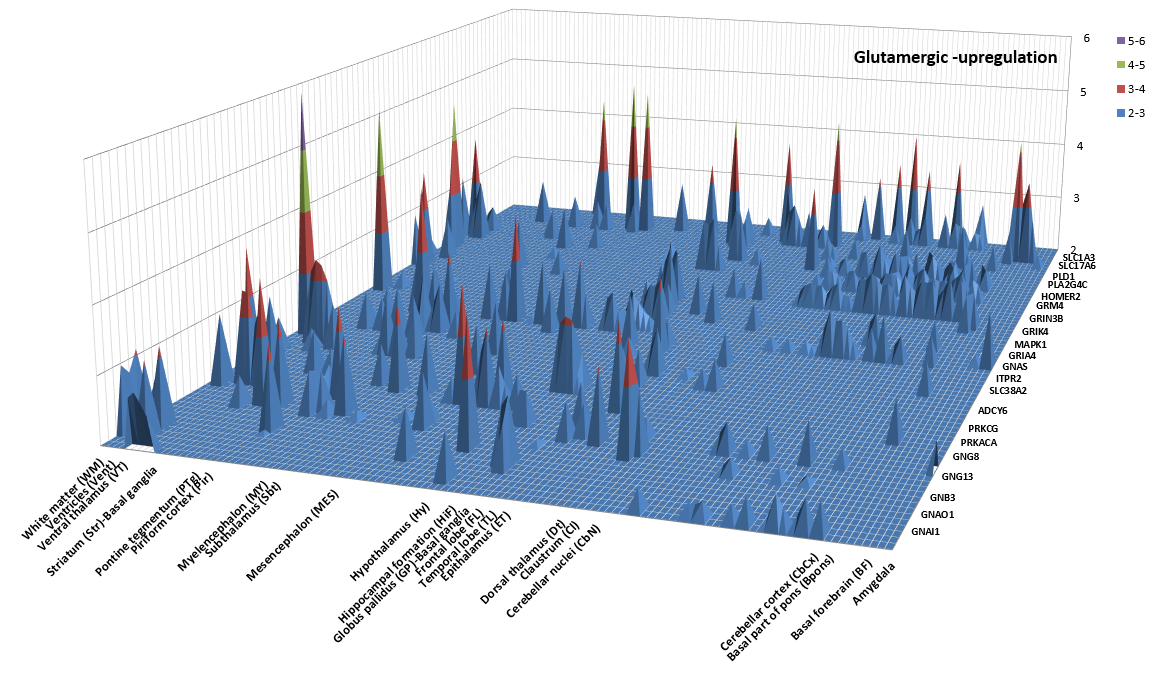
**

**Supplementary Figure S4c.** Structural distribution of gene expression in the glutamergic neurotransmitter systems

**
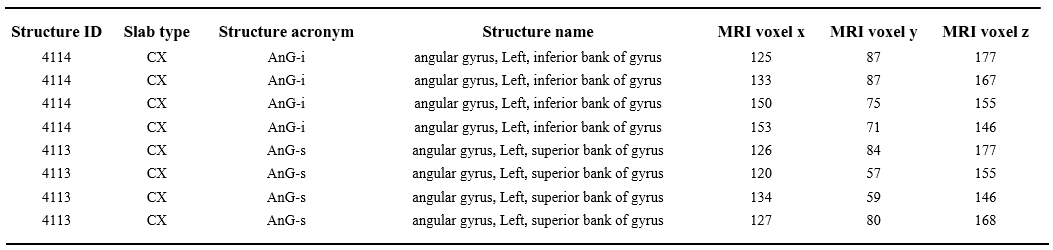
**

**Supplementary Figure S5.** Illustration of microarray sampling sites mapped into each donor’s MR image space. The gene expression was averaged for identically annotated microarray sampling sites

**
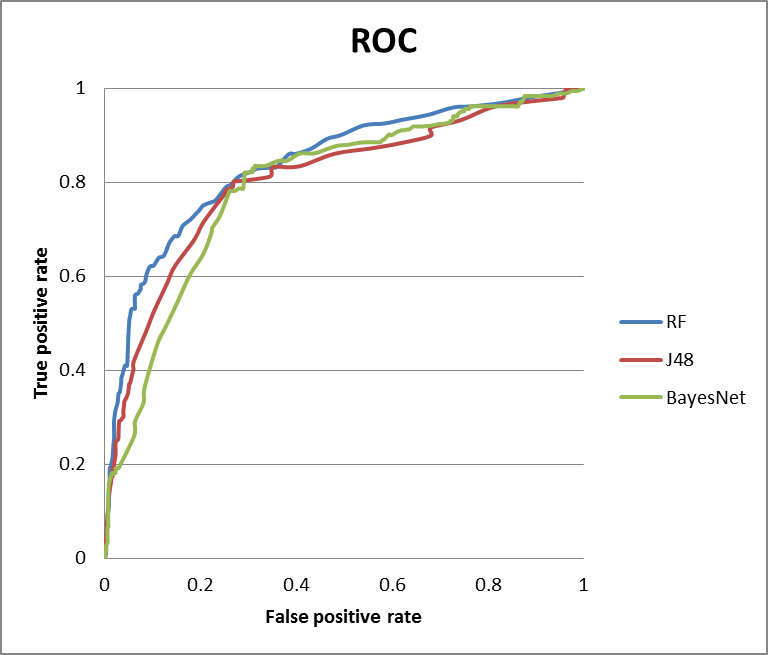
**

**Supplementary Figure S6**. ROC curves showing the relationship between TPR (sensitivity) and FPR (1-specificity) for the three ML algorithms used for model building to predict genes associated with Parkinson’s disease. We obtained an AUC of 0.82 by RF (Random Forest), which indicates that the classification model can differentiate between the Parkinson’s versus non-Parkinson’s associated genes efficiently.
